# Activity of Temporin A and Short Lipopeptides Combined with Gentamicin against Biofilm Formed by *Staphylococcus* *aureus* and *Pseudomonas aeruginosa*

**DOI:** 10.3390/antibiotics9090566

**Published:** 2020-09-02

**Authors:** Malgorzata Anna Paduszynska, Katarzyna Ewa Greber, Wojciech Paduszynski, Wieslaw Sawicki, Wojciech Kamysz

**Affiliations:** 1 Department of Inorganic Chemistry, Faculty of Pharmacy, Medical University of Gdansk, 80-416 Gdańsk, Poland; kamysz@gumed.edu.pl; 2 Department of Physical Chemistry, Faculty of Pharmacy, Medical University of Gdansk, 80-416 Gdańsk, Poland; katarzyna.greber@gumed.edu.pl (K.E.G.); wieslaw.sawicki@gumed.edu.pl (W.S.); 3 Department of Surgery, Poviat Hospital in Bytow, 77-100 Bytow, Poland; wojpad@wp.pl

**Keywords:** lipopeptides, antimicrobial peptides, gentamicin, biofilm-associated infections, microbial resistance, combination therapy, synergism

## Abstract

The formation of biofilms on biomaterials causes biofilm-associated infections. Available treatments often fail to fight the microorganisms in the biofilm, creating serious risks for patient well-being and life. Due to their significant antibiofilm activities, antimicrobial peptides are being intensively investigated in this regard. A promising approach is a combination therapy that aims to increase the efficacy and broaden the spectrum of antibiotics. The main goal of this study was to evaluate the antimicrobial efficacy of temporin A and the short lipopeptides (C10)2-KKKK-NH2 and (C12)2-KKKK-NH2 in combination with gentamicin against biofilm formed by Staphylococcus aureus (SA) and Pseudomonas aeruginosa (PA). Peptides were synthesized with solid-phase temperature-assisted synthesis methodology. The minimum inhibitory concentrations (MICs), fractional inhibitory concentrations (FICs), minimum biofilm eradication concentrations (MBECs), and the influence of combinations of compounds with gentamicin on bacterial biofilm were determined for reference strains of SA (ATCC 25923) and PA (ATCC 9027). The peptides exhibited significant potential to enhance the antibacterial activity of gentamicin against SA biofilm, but there was no synergy in activity against planktonic cells. The antibiotic applied alone demonstrated strong activity against planktonic cells and poor effectiveness against SA biofilm. Biofilm formed by PA was much more sensitive to gentamicin, but some positive influences of supplementation with peptides were noticed. The results of the performed experiments suggest that the potential application of peptides as adjuvant agents in the treatment of biofilm-associated infections should be studied further.

## 1. Introduction

Biofilm-associated infections are a serious problem in modern medicine. They usually occur as complications of common medical procedures involving the application of biomaterials such as cardiac implants, catheters and vascular and orthopedic prostheses [1]. The most common etiological factors of biofilm-associated infections include *Staphylococcus aureus* (SA), *Staphylococcus epidermidis* (SE), *Enterococcus faecalis* (EF), *Streptococcus viridans* (SV), *Escherichia coli* (EC), *Klebsiella pneumoniae* (KP), *Proteus mirabilis* (PM) and *Pseudomonas aeruginosa* (PA) [2,3,4]. It is very likely that the bacterial strain does not show resistance to antibiotics in the planktonic form, while the same antibiotics fail to fully eradicate bacteria living as a biofilm [5]. The infections tend to return after withdrawal of the antibiotics or become chronic, which poses a serious threat to the patient’s health and life. Due to lack of sufficient treatments for biofilm-associated infections, there is an urgent need to develop new active agents and/or strategies to solve this problem.

One intensively investigated group of compounds is antimicrobial peptides (AMPs). AMPs have been identified in numerous living organisms including plants, insects, amphibians and mammals in variety of tissues and mucosal surfaces exposed to the external environment, where they are considered to play a crucial role in defense from microbial infection [6,7,8,9,10]. AMPs demonstrate antimicrobial activity towards Gram-positive and Gram-negative bacteria as well as fungi and viruses [11,12,13,14]. Besides direct antimicrobial action, they can also boost the adaptive immune response to further manage infection, neutralize the action of lipopolysaccharide (LPS) and play a crucial role in wound-healing processes [15,16,17]. Numerous studies demonstrate their activity against drug-resistant pathogens, effectiveness against biofilms and low rate of resistance development. Due to these properties, they are considered a promising group of antimicrobials. However, there are several limitations for their therapeutic applications. The most significant ones are the relatively low bioavailability and high costs of production [18,19].

As the development of microbial resistance exceeds the development of new infallible antibacterials, combination therapy has gained the attention of numerous researchers. This involves using two or more antimicrobials together to restore or increase their efficacy against the resistant pathogen. Combination therapy aims to broaden the spectrum of activity and/or increase the efficacy of antibiotics, which allows the applied dosage and potential side effects to be reduced [20].

Gentamicin is an aminoglycoside antibiotic, which binds with small ribosomal subunits to inhibit the synthesis of bacterial proteins. Its broad spectrum of bactericidal activity includes gram-positive bacteria. The antibiotic is applied in many branches of medicine. It is widely used in topical therapy of dermatological and ocular infections. Gentamicin is administered parenterally in the treatment of intra-abdominal infections, urinary tract infections, respiratory tract infections, secondary infections of burns and traumatic and postoperative wounds and severe infections in newborns. In the form of a collagen-bound implant, gentamicin is used in supportive treatment and the prevention of bacterial infections of bones and soft tissues (e.g., bone grafts, artificial joint implants fixed with a cementless technique). Side effects of gentamicin are associated with its nephrotoxicity, neurotoxicity, ototoxicity and gastrointestinal complications, and occur mainly after its parenteral administration [21].

Microbial resistance to gentamicin is developing slowly and constantly. This concerns, among others, Enterobacteriaceae, *Pseudomonas* spp., *Enterococcus* spp., *Staphylococcus* spp. [22]. A cross resistance with other aminoglycosides is possible, while synergistic action towards some bacteria was confirmed for penicillins and cephalosporins [21]. The antibiotic demonstrates synergistic activity with ciprofloxacin and fosfomycin against biofilms formed by Gram-negative bacteria [23].

The goal of the presented work was to evaluate the antibiofilm potential of gentamicin in combination with an amphibian AMP temporin A (TA) and two short lysine-based synthetic lipopeptides containing two fatty acid residues: (C10)2-KKKK-NH2 (lipopeptide 1, L-1) and (C12)2-KKKK-NH2 (lipopeptide 2, L-2). TA is a highly hydrophobic frog-derived AMP, isolated from Rana temporaria. It exhibits activity against Gram-positive bacteria including antibiotic-resistant Gram-positive cocci through increasing the microbial membrane permeability. An increase of TA activity in combination with other antimicrobials has been found [24,25].

Short lipopeptides are compounds designed to imitate the features of positively charged, amphipathic AMPs, but are less cost- and time-consuming in production [26,27]. The combinations of compounds were tested against biofilms formed by SA and PA formed on polystyrene surfaces.

## 2. Results

Gentamicin demonstrated high activity (MIC < 1 mg/L) against both tested strains cultured in a liquid medium (Table 1). TA was active against SA at a concentration of 8 mg/L, while to inhibit the growth of PA, the compound had to be applied at the highest concentration tested. The short lipopeptides were active against both strains. The obtained MICs were 8 and 16 mg/L against SA, and 16 and 32 against PA for L-1 and L-2, respectively (Table 1).

The obtained MIC values of combined compounds were equal to the MIC values of compounds alone, which indicates no synergy at the concentrations tested (data not shown). In the case of TA for both strains and L-1 for SA, the MICs of combined compounds were equal to 1/2 of MICs of compounds alone. However, the reduction of active concentration by half is not sufficient to classify the combined compounds as synergistic, and the interaction is classified as indifferent (Table 1) [20].

**Figure 1:**
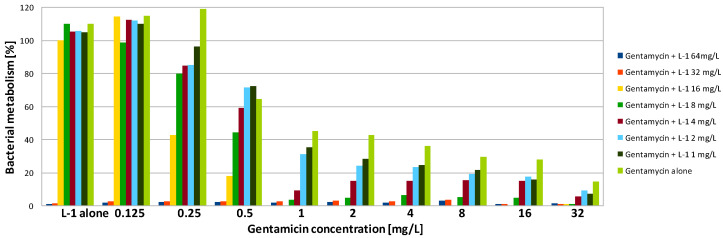
Activity of gentamicin in combination with lipopeptide 1 (L-1) against SA biofilm. Results presented as a percentage of metabolic activity in comparison to positive (100%) and negative (0%) controls; RSD ≤ 20%.

**Figure 2:**
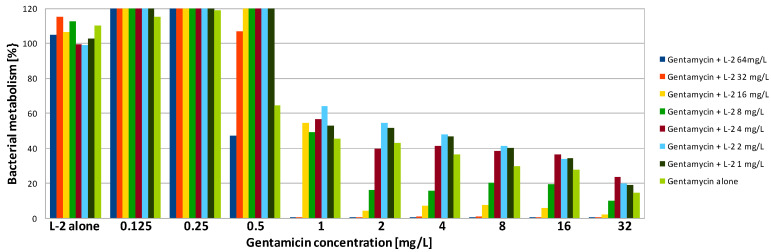
Activity of gentamicin in combination with lipopeptide 2 (L-2) against SA biofilm. Results presented as percentage of metabolic activity in comparison to positive (100%) and negative (0%) controls; RSD ≤ 20%.

**Figure 3:**
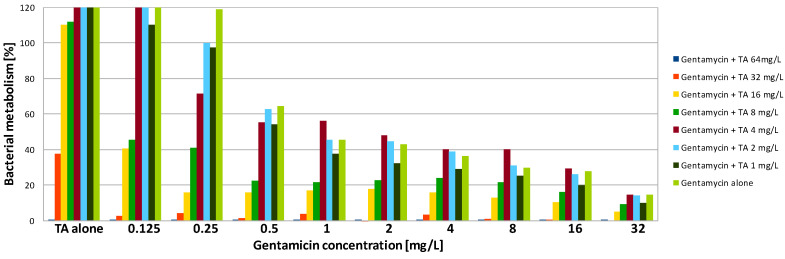
Activity of gentamicin in combination with temporin A (TA) against SA biofilm. Results presented as percentage of metabolic activity in comparison to positive (100%) and negative (0%) controls; RSD ≤ 20%.

Gentamicin was highly active against biofilm formed by PA on a polystyrene surface. Both MBEC90 and MBEC50 were only 2 mg/L, which was four times higher compared to the MIC (Table 2). In contrast, SA formed structures showing a high level of resistance to the antibiotic. Application of gentamicin at the highest tested concentration (32 mg/L—128 times higher than the MIC) was not sufficient to reduce the metabolism of SA cells to 10%. However, some activity was observed—after exposure of SA biofilm to gentamicin at concentrations of 32 and 16 mg/L, metabolism was reduced to ~15% and 30%, respectively, while to reduce it by half, a concentration of 1 mg/L was sufficient (Figure 1, Figure 2 and Figure 3, Table 2).

**Figure 4:**
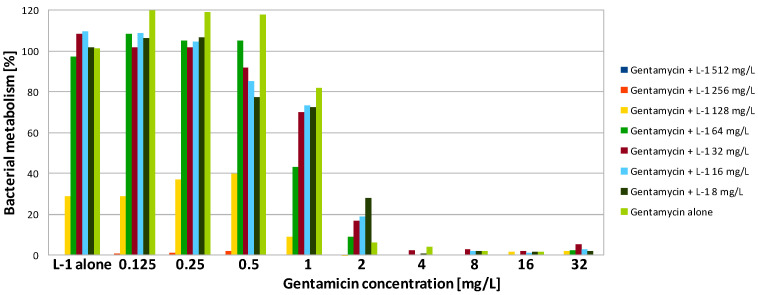
Activity of gentamicin in combination with lipopeptide 1 (L-1) against PA biofilm. Results presented as percentage of metabolic activity in comparison to positive (100%) and negative (0%) controls; RSD ≤ 20%.

**Figure 5:**
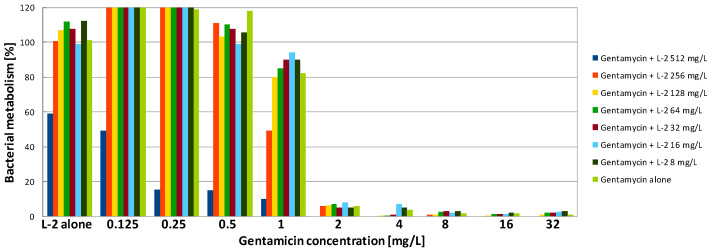
Activity of gentamicin in combination with lipopeptide 2 (L-2) against PA biofilm. Results presented as percentage of metabolic activity in comparison to positive (100%) and negative (0%) controls; RSD ≤ 20%.

**Figure 6:**
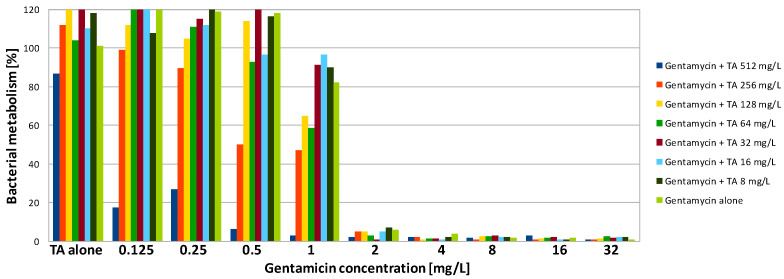
Activity of gentamicin in combination with temporin A (TA) against PA biofilm. Results presented as percentage of metabolic activity in comparison to positive (100%) and negative (0%) controls; RSD ≤ 20%.

In contrast to gentamicin, AMPs demonstrated poor activity against PA biofilm. The highest, but still rather poor, activity was presented by L-1. The obtained MBEC90 value was 256 mg/L, which was 16 times higher than the MIC (Table 2). Exposure to the peptide at 128-mg/L reduced PA metabolism to 30% (Figure 4). L-2 demonstrated very weak activity. Application of the peptide at the highest concentration (512 mg/L) was insufficient to reduce the metabolism by half (Table 2). Exposure of PA biofilm to the peptide at 512 mg/L resulted in metabolism reduction to 60% (Figure 5). A total lack of activity of TA was expected, as the peptide was also not active against the planktonic culture (Figure 6, Table 2). SA cultured on polystyrene was much more sensitive to AMPs. To obtain a 90% reduction of metabolism, TA and L-1 were applied at 64 and 32 mg/L, respectively. Both peptides reduced the SA metabolism by half when applied at a concentration of 32 mg/L, which was four times higher than the MIC in both cases (Table 2). L-2 applied alone had no influence on SA biofilm (Figure 2).

The combination of gentamicin with all tested peptides resulted in a significant increase in effectiveness of the compounds against biofilm formed by SA. As the value of MBEC90 was not determined at a tested concentration range, it is not possible to calculate the fractional biofilm eradication concentration index (ΣFBEC) in order to classify the interaction. However, the obtained results show that there is an obvious positive interaction between gentamicin and peptides. To reduce the SA metabolism to at least 10% using a combination of gentamicin with L-1, the compounds were used at a concentration of 1 and 4 mg/L, respectively (Table 3). The application of the compounds together allowed the concentration of L-1 to be four times lower, while for gentamicin, the active concentration was 32 times lower (Figure 1). The same concentration of antibiotic had to be applied when it was used with L-2. L-2 had to be applied at 32 mg/L (Figure 2), while when applied alone, it was not active at the highest tested concentration (64 mg/L). The application of gentamicin with TA allowed the concentration of the antibiotic to be reduced to 0.125 mg/L, which was even lower than the MIC obtained for planktonic bacteria. The peptide was applied at a concentration of 32 mg/L, which was two times lower than the obtained MBEC90 (Figure 3). As expected, the combination of gentamicin and peptides resulted as well in a decrease of the determined MBEC50 values (Table 4, Figure 1, Figure 2 and Figure 3).

In the case of PA cultures, there was no obvious interaction between the peptides and gentamicin. However, some reduction in both MBEC90 and MBEC50 values was observed (Table 3 and Table 4). The application of the antibiotic with both lipopeptides allowed the concentration of gentamicin to be reduced by half. The active concentration of L-1 in combination was also two times lower than the MBEC90 and MBES50 determined for the compound alone (Figure 4). The ΣFBEC was equal to 1, so the interaction is classified as indifferent. In the case of L-2, its combination with gentamicin was effective against PA biofilm when the compound was used at a concentration of 512 and 256 mg/L for MBEC90 and MBEC50, respectively (Figure 5). The lipopeptide was not active against PA biofilm when tested alone, so there was a stronger interaction compared to L-1. The application of gentamicin with TA allowed the active concentration of the antibiotic to be four times lower. The peptide was applied at 512 and 256 mg/L in order to reduce bacterial metabolism to 10 and 50%, respectively (Figure 6). It is worth noting that TA, once applied at the concentration of 512 mg/L, was not active against PA even in the planktonic culture.

Interestingly, exposure of SA and PA biofilm to gentamicin and peptides at low concentrations resulted in some increase of metabolic activity than the positive control (Figure 1, Figure 2, Figure 3, Figure 4, Figure 5 and Figure 6). This may be explained by the defense mechanism of bacteria from the hostile environment. Biofilm formation allows bacteria to avoid the action of human AMPs and antibiotics in a human body [5].

## 3. Discussion

The use of medical devices and implants significantly improves therapeutic options, but it also creates the risk of biofilm-associated infections [28,29]. The standard means of treatment for these infections are very often insufficient. Numerous native AMPs and their derivatives, such as short lipopeptides, have been investigated for their potential application in the prophylaxis and treatment of such infections [28,30,31,32,33,34,35,36].

The results obtained for the compounds applied alone confirm the available data that reports strong activity of AMPs against SA biofilm. TA and L-1 applied at the determined MBEC90 caused a reduction of bacterial metabolism to ~1%. The MBECs90 were only few times higher than the MICs. Gentamicin did not cause a sufficient reduction of biofilm even when applied at a concentration over 100 times higher than the MIC. It has previously been reported that MBECs determined for conventional antimicrobials were at least 50–100 times higher than MICs [37,38]. Discrepancies between the activity of compounds against planktonic cells and biofilms are associated with the presence of extracellular polymeric substances (EPS), which protect microbial cells from a hostile environment, changes of the gene expression, slowing down metabolism and the development of persister cells, which repopulate the culture after the withdrawal of the antimicrobial agent [5,39]. In a previous study, L-1 demonstrated the ability to permanently eliminate the biofilms of Gram-positive bacteria, while in the case of conventional antimicrobials, after their withdrawal from the culture medium, the bacteria repopulated to a high extent [40]. The antistaphylococcal activities of numerous lipopeptides were confirmed on larger sets of SA strains, including antibiotic-resistant clinical isolates as well as bacteria cultured in the form of biofilm [37,38,40,41,42,43,44,45,46]. In a previous study, TA and another amphibian peptide, citropin 1.1, were effective against biofilms formed by staphylococci and streptococci resistant to erythromycin, gentamicin and neomycin. Moreover, in contrast to conventional antimicrobials, their activity did not depend on the time of biofilm cultivation [38]. Promising results obtained for AMPs are associated with their ability to kill slow or even non-growing, bacteria and the small size of the molecules, which enables penetration through EPS [47]. Surprisingly, in this study L-1 showed no influence on SA biofilm, while its activity against planktonic cells was only twice lower than L-1 and TA. Gentamicin was much more active against planktonic cells in comparison to the AMPs, however the obtained MICs against SA allow all tested peptides to be defined as highly active according to the classification presented by Shai and Abrahami [48].

Both lipopeptides were active against the planktonic cells of PA, while TA demonstrated very weak activity. All peptides exhibited poor activity towards the PA biofilm, while gentamicin was highly active against PA in planktonic cells as well as in the biofilm. Other lipopeptides as well as amphibian peptides also showed poor activity against biofilm formed by Gram-negative strains, while conventional antimicrobials demonstrated much higher activity [38,40,41]. However, some data reports the resistance of PA biofilms to even highly active compounds like ciprofloxacin and fosfomycin [49]. Due to the excessive resistance mechanisms and biofilm formation, PA is a critical human pathogen that causes life-threatening infections, including hospital-acquired pneumonia, ventilator-associated pneumonia and bloodstream infections. PA is often isolated from cystic fibrosis (CF) patients, where is it responsible for chronic infection and constitutes a significant cause of morbidity and mortality in CF patients [50].

In our study, gentamicin was highly active against biofilm formed by PA. However, some enhancement of activity was observed after the application of the antibiotic in combination with the AMPs. Surprisingly, this was shown for compounds that displayed no activity against PA biofilm once applied alone. Moreover, the compounds did not show any interaction against PA in planktonic form. L-1 has shown the ability to increase the effectiveness of contact lens solutions against PA biofilm formed on contact lenses, which was the most difficult biofilm to eliminate from the surface of contact lenses using contact lens solutions, amphibian peptides and short lipopeptides containing hexadecanoic acid [40,51]. Polymyxin B and colistin—AMPs that are used in medicine—have shown synergistic activity with fluoroquinolones against PA biofilm [50]. Ruden et al. presented a high number of active combinations against a multidrug resistant (MDR). *Pseudomonas aeruginosa* isolate (PA910). Tests for interaction with conventional antimicrobials, including gentamicin, have been conducted for 30 short AMPs from different origins. The antibiotic showed a synergist interaction with novel variants of indolicidin [52].

Gentamicin demonstrates synergy with several compounds of different origin against a broad spectrum of microorganisms, e.g., *Acinetobacter* baumannii, EC and SA [53,54,55]. As mentioned before, SA is one of the most common etiological factors of biofilm-associated infections. Moreover, the resistance of SA to conventional antimicrobial therapy is growing rapidly and constantly. For these reasons, numerous studies focus on the development of effective strategies to fight MDR SA and SA biofilm.

The results of current work confirm the high activity of AMPs against SA biofilm. Moreover, the combination of gentamicin with all tested peptides further improved the antibiofilm potential. The determined active concentrations of compounds decreased substantially when they were used in combinations. Interestingly, such strong interactions between compounds against biofilm were observed while there was no synergistic effect against planktonic cultures of SA.

A combinatorial antibiotic application with AMPs has been found to be more effective in comparison with monotherapy in previous studies. The lipopeptide C16-KK-NH2 enhanced the activity of vancomycin used in the prophylaxis of vascular graft infection in the rat model [56]. The amphibian peptide citropin 1.1 demonstrated synergistic action with rifampin and minocycline against a SA biofilm [57]. Derivatives of *P*-113 created by substitution of non-natural amino acid residues restored the antimicrobial activity of vancomycin against vancomycin-resistant *E. faecium*, SA and wild-type EC [58].

Ciandrini et al. reported synergy between AMPs used in combinations against MRSA biofilms. Synergistic action of AMPs is expected, as they are present in living organisms as sets of peptides produced in various amounts, and their biologic action results from joint activities. However, the authors applied peptides of different origin: citropin 1.1 and TA from different species of amphibians, a hybrid peptide of peptides from insects— cecropin A and melittin—and a representative of short lipopeptides: C16-KGK-NH2. These findings are very promising with regard to the development of novel AMP-based antistaphylococcal therapies. The application of peptides in co-therapy allowed the applied concentrations to be significantly reduced [59].

In the current work, application of gentamicin with L-1 reduced the active concentrations over 32 and 4 times, respectively. Cotreatment with TA allowed the amount of gentamicin to be further reduced (over 128 times), while the peptide concentration was two times lower. Beyond the improved efficacy of compounds, a combination therapy also offers the possibility to reduce toxicity and the potential side effects of compounds due to the application of lower dosages.

The main limitation of the therapeutic application of many AMPs is their high toxicity resulting from their nonspecific mechanism of action. The compounds disrupt the membranes of red blood cells when the cells are exposed to the compounds at concentrations close to their MICs [27]. Due to high toxicity in vitro, the potential application of lipopeptides containing hexadecanoic acid, which present excellent antibiofilm activities, is limited to topical skin application [37,38,45,60]. The results obtained in a previous study revealed high toxicity of palmitic acid derivatives, which suggests their non-selective membrane activity [60]. TA exhibits a negative influence on the proliferation and viability of HaCaT keratinocytes at a concentration of 50 mg/L [45]. The application of TA with gentamicin allows the MBEC against SA of the peptide to be reduced from 64 to 32 mg/L, which is determined as safe to human cells. The lipopeptides with two residues of decanoic and dodecanoic acid also show cytotoxicity towards HaCaT cells at a concentration of ~50 mg/L. The use of lipopeptides with gentamicin is an effective antistaphylococcal combination, where peptides are applied at concentrations nontoxic towards HaCaT keratinocytes as well as human red blood cells [60,61].

## 4. Materials and Methods

### 4.1. Bacterial Strains and Culture Conditions

SA (ATCC 25923) and PA (ATCC 9029) were purchased from the Polish Collection of Microorganisms (Polish Academy of Science, Wroclaw, Poland). The bacteria were suspended in Mueller–Hinton Broth II (MHB II, Biocorp, Warsaw, Poland) and incubated under aerobic conditions at 37 °C. After 24 h of incubation, the cultures were centrifuged (10 min, 2500 rpm), washed three times with phosphate buffer saline (PBS, AppliChem, Darmstadt, Germany) and suspended in MHB II to the appropriate inocula.

### 4.2. Antimicrobials

Gentamicin sulfate was purchased from Sigma-Aldrich. Lipopeptides (C10)2-KKKK-NH2, (C12)2-KKKK-NH2 and TA were synthesized manually using the solid-phase synthesis method. The peptide bond was created according to the previously described protocol [41].

### 4.3. Minimum Inhibitory Concentration (MIC)

The MICs of AMPs and gentamicin were determined on SA and PA using the broth dilution method protocol recommended by the Clinical and Laboratory Standard Institute (CLSI) [62]. Suspensions of bacteria in MHB II at inocula of ~5 × 106 CFU (colony forming units)/mL were added to 96-well spherical bottom polystyrene plates (PS, Kartell, Noviglio, Italy) and exposed to solutions of peptides (concentration range: 1–64 mg/L for SA and 8–512 mg/L for PA) and gentamicin (concentration range: 0.125–32 mg/L) in MHB II. The samples were incubated for 18h under aerobic conditions at 37 °C. The MIC (mg/L) was taken as the lowest concentration of the antimicrobial that inhibited the visible growth of the bacteria. All experiments were performed in triplicate and included growth and sterility controls.

### 4.4. Fractional Inhibitory Concentration (FIC)

FIC was determined with the checkerboard assay. Suspensions of bacteria in MHB II at inocula of ~5 × 106 CFU/mL were added to 96-well spherical bottom PS (Kartell, Noviglio, Italy) and exposed to gentamicin combined with AMPs at increasing concentrations. Two-fold dilutions of compounds were distributed to the wells (gentamicin: 0.125–32 mg/mL; peptides: 1–64 mg/L for SA and 8–512 mg/L for PA). Gentamicin was serially diluted along the plate, while the peptides were diluted from top to bottom. The samples were incubated under aerobic conditions at 37 °C for 24 hours. The lowest concentration of the combined compounds that inhibited the visible growth of bacteria was considered as the effective MIC for the combination.

The fractional inhibitory concentration index (ΣFIC) was calculated according to the formula below:

ΣFIC = FIC A + FIC B,

where:

FIC A = MIC of antimicrobial A in the combination/MIC of antimicrobial A alone

FIC B = MIC of antimicrobial B in the combination/MIC of antimicrobial B alone

The combination was classified as synergistic when the ΣFIC was ≤0.5. Indifference was indicated by a ΣFIC >0.5 to ≤ 4 and antagonism was indicated when the ΣFIC was >4 [20].

### 4.5. Minimum Biofilm Eradication Concentration (MBEC)

Suspensions of SA and PA at initial inocula of ~5 × 108 CFU/mL in MHB II were added to the plates and incubated under aerobic conditions, shaking (120 rpm) at 37 °C for 24 h. The wells were then washed three times with PBS and MHB II supplemented with serial dilutions of gentamicin (range: 32–0.125 mg/L) and AMPs (range: 64–0.5 for SA and 512–4 mg/L) was added. After 24 h of exposure (aerobic conditions, 120 rpm shaking, 37 °C), the wells were washed three times with PBS and a solution of 0.01% resazurin (Sigma-Aldrich, St. Louis, MO, USA) in MHB II was added. Resazurin is a cell-viability reagent that is reduced by dehydrogenases of living bacteria to a pink resorufin. After 1 h of incubation, the absorbance was measured at 570 and 600 nm with a microplate reader (Thermo Fisher Scientific, Waltham, MA, USA). The% of metabolic activity (MA) when compared with positive (sample with bacteria suspended in pure MHB II) and negative (pure MHB II) controls, which were taken as 100% and 0%, respectively, was calculated according to the following formula:

MA(%) = (ΔAbs of sample − ΔAbs of negative control)/(ΔAbs of positive control − ΔAbs of negative control);

ΔAbs = absorbance at 570 nm– absorbance at 600 nm.

The results are presented as MBEC90 and MBEC50, which were taken as the lowest concentrations of antimicrobials that reduced the MA of bacteria by at least 90 ± 5% and 50 ± 5%, respectively. The MA taken for determining the MBEC were the means of three results obtained on three different days.

### 4.6. Activity of Gentamicin Applied in Combination with AMPs against Biofilms Formed by SA and PA on 96-well PS

Biofilms cultured on PS (as described in Section 4.5) were exposed to combinations of gentamicin with lipopeptides and TA. Two-fold dilutions of compounds were distributed to the wells (gentamicin: 0.125–32 mg/mL; peptides: 1–64 mg/L for SA and 8–512 mg/L for PA). Gentamicin was serially diluted along the plate, while the peptides were diluted from top to bottom. The samples were incubated for 24 h (aerobic conditions, 120 rpm shaking, 37 °C). After exposure, the wells were washed three times with PBS and a solution of resazurin was added. The results were read and calculated as described in Section 4.5. The results were presented as a% of living cells (MA) when compared with positive (sample with bacteria suspended in pure MHB II) and negative (pure MHB II) controls, which were taken as 100% and 0%, respectively. The presented results are the means of three results obtained on three different days.

The fractional biofilm eradication concentration index (ΣFBEC) was calculated according to the formula below:

ΣFBEC = FBEC A + FBEC B,

where:

FBEC A = MBEC of antimicrobial A in the combination/MBEC of antimicrobial A alone;

FBEC B = MBEC of antimicrobial B in the combination/MBEC of antimicrobial B alone.

The combination was classified as synergistic when the ΣFBEC was ≤0.5. Indifference was indicated by a ΣFBEC >0.5 to ≤ 4 and antagonism was indicated when the ΣFBEC was >4.

## 5. Conclusions

The results obtained in the present study revealed that TA and the lipopeptides (C10)2-KKKK-NH2 and (C12)2-KKKK-NH2, when combined with gentamicin, exhibit strong synergistic action towards SA biofilm. Considering the fact that staphylococci are one of the most frequent causes of biofilm-associated infections, these findings are very promising, and the research should be continued on clinical strains. Due to the lack of synergy between compounds against planktonic cells, the search for combinations effective against biofilm should include compounds that do not show synergy in the FIC assay. AMPs are definitely very interesting candidates as effective adjuvant agents for standard therapy of biofilm-associated infections. However, in addition to the extension of microbiological studies, numerous other issues like toxicity, absorbance, metabolism, and the elimination of combinations still need to be researched in order to ensure patient safety.

## Figures and Tables

**Table 1:** Activity against planktonic bacteria: minimum inhibitory concentration (MIC) (mg/L) and fractional inhibitory concentration index (ΣFIC) determined for gentamicin and antimicrobial peptides against *Staphylococcus aureus* (SA) and *Pseudomonas aeruginosa* (PA).

Compound	SA			PA		
	MIC	ΣFIC with Gentamicin	Interaction	MIC	ΣFIC with Gentamicin	Interaction
Gentamicin	0.25	–	–	0.5	–	–
Temporin A	8	1	indifferent	512	1	indifferent
Lipopeptide 1	8	2	indifferent	16	2	indifferent
Lipopeptide 2	16	1	indifferent	32	2	indifferent

**Table 2:** Minimum biofilm eradication concentration (MBEC) (mg/L) determined for gentamicin and antimicrobial peptides against SA and PA.

Compound	MBEC (mg/L)					
	SA			PA		
	MBEC90	MBEC50	Enhancement of Activity of Gentamicin	MBEC90	MBEC50	Enhancement of Activity of Gentamicin
Gentamicin	>32	1	–	2	2	–
Temporin A	64	32	+++	>512	>512	++
Lipopeptide 1	32	32	+++	256	128	+
Lipopeptide 2	>64	>64	+++	>512	>512	+

**Table 3:** MBEC90 (mg/L) obtained for combinations of gentamicin and antimicrobial peptides against SA and PA.

Compound	MBEC 90 (mg/L)			
	SA		PA	
	Gentamicin	Peptide	Gentamicin	Peptide
Gentamicin alone	>32	–	2	–
Lipopeptide 1 alone	–	32	–	256
Gentamicin + L-1	1	4	1	128
Lipopeptide 2 alone	–	>64	–	>512
Gentamicin + L-2	1	32	1	512
Temporin A alone	–	64	–	>512
Gentamycin + TA	0.125	32	0.5	512

**Table 4:** MBEC 50 (mg/L) obtained for combinations of gentamicin and antimicrobial peptides against SA and PA.

Compound	MBEC 50 (mg/L)			
	SA		PA	
	Gentamicin	Peptide	Gentamicin	Peptide
Gentamicin alone	1	–	2	–
Lipopeptide 1 alone	–	32	–	128
Gentamicin + L-1	0.5	8	1	64
Lipopeptide 2 alone	–	> 64	–	> 512
Gentamicin + L-2	0.5	64	1	256
Temporin A alone	–	32	–	> 512
Gentamicin + TA	0.125	8	0.5	256
